# Functional Foods Acceptability: A Consumers’ Survey on Bread Enriched with Oenological By-Products

**DOI:** 10.3390/foods12102014

**Published:** 2023-05-16

**Authors:** Roberta Miolla, Giovanni Ottomano Palmisano, Rocco Roma, Francesco Caponio, Graziana Difonzo, Annalisa De Boni

**Affiliations:** Department of Soil, Plant and Food Sciences (DISSPA), University of Bari Aldo Moro, 70126 Bari, Italy; roberta.miolla@uniba.it (R.M.); rocco.roma@uniba.it (R.R.); francesco.caponio@uniba.it (F.C.); graziana.difonzo@uniba.it (G.D.);

**Keywords:** oenological by-products, functional foods, circular economy, consumers’ acceptance, cluster analysis

## Abstract

In recent years, consumers have shown considerable attention to functional foods that can provide various benefits. At the same time, the awareness of the problem of waste generation from the agri-food supply chains has increased; thus, scholars and practitioners are devoting great attention to sustainable food waste management. Within the wine processing, the production phase generates by-products such as marc, grape seeds, stems, and wine lees. In most cases, these by-products are treated as waste rather than as a resource, creating environmental, economic, and social impacts related to their disposal. By contrast, the reuse of oenological by-products in food production can have several health benefits, since they are rich in functional molecules such as fibres, polyphenols, and vitamin E, and can also trigger a circular economy model. The aim of this research is to investigate the acceptance of consumers towards bread enriched with oenological by-products through the application of k-means clustering, providing insights on the characterisation of groups of consumers based on their specific features and declared attitudes. The results showed three different consumers’ clusters, highlighting that the acceptance of this enriched bread is not influenced by the consumers’ socio-economic features, but it is related to consumers’ sensitivity. Therefore, target strategies should be put in place to inform consumers about the benefits associated with the consumption of bread enriched with oenological by-products.

## 1. Introduction

The linear economy is the most widespread model focused on the conventional paradigm system of the take–make–dispose [1]. The linear food system, focused on the procurement of raw materials, processing, and waste disposal [2], is acknowledged as wasteful, polluting, and depletive, and is responsible for one half of the loss of biodiversity and for one third of global greenhouse gas emissions [3]. The current food system, while allowing the feeding of the growing population, it, however, does not allow the economy to follow a sustainability-oriented approach [4]. Therefore, the European Commission proposed the following definition for circular economy (CE): “in a CE, the value of products and materials is maintained for as long as possible; waste and resource use are minimised, and resources are kept within the economy when a product has reached the end of its life, to be used again and again to create further value” [5]. This stresses the need to convert the linear food systems into a defined circular economy food model, enhancing the by-products thanks to their introduction into the production cycle while also reducing the environmental impact caused by industries [5,6]. This need arises from a perspective of the valorisation of waste and by-products derived from the food production process. Indeed, the agri-food supply chain represents one of the hotspots generating great amounts of waste and pollutants affecting the environment [7].

For many years, the issues related to the disposal of waste and agri-food by-products have generated concerns both for companies affected by the economic impact of waste management and disposal, and for society alarmed by the environmental impacts [8]. However, insights from the literature [9,10,11] showed a growing interest of consumers towards the health effects of foods enriched with a high nutritional value, such as antioxidants, polyphenols, fibres, minerals, and vitamins. In this regard, the by-products derived from the wine industry can represent an optimal source from which to recover ingredients with high added value [12]. In fact, the numerous beneficial effects that the bioactive molecules present in wine by-products exert could be exploited by introducing them into the production cycle of various foods to obtain benefits for the human body. Traditional bread is a staple in Mediterranean countries and in the Italian diet, as it accounts for almost 40% of the total cereal intake [13,14]. According to Gil et al. [15], foods based on wholegrain cereals, including bread, play a crucial role in human health and well-being. Indeed, wholegrain cereals are rich in nutrients and phytochemical compounds with recognised benefits for health, and their regular consumption reduces the risk of cardiovascular diseases, type 2 diabetes mellitus, and certain types of cancer, as well as several gastrointestinal pathologies. Bread added with plant-based food by-products is defined as “functional bread”. The incorporation of these by-products in bread is a viable way of improving further the health and nutritional status of consumers and reducing food waste [16,17].

The aim of this research is to investigate the acceptance of Italian consumers towards bread enriched with oenological by-products, through data collection with a tailored questionnaire and the subsequent application of k-means clustering. This will advance the knowledge on the acceptance of food enriched with by-products, and particularly will provide insights on the characterisation of groups of consumers based on their specific features and declared attitudes for the consumption of bread enriched with oenological by-products.

The paper is structured as follows: After the analysis of the literature review in Section 2, Section 3 describes the materials and methods. Then, Section 4 and Section 5 report the results and discussion. Finally, conclusive remarks are highlighted in Section 6.

## 2. Literature Review

### 2.1. Oenological By-Products and Their Functional Compounds

Oenological by-products include grape marc, wine lees, grape stalks, grape seeds, and vine shoots. Grape marc, consisting of pulp, skins, stalks remnant and grape seeds, derives from the crushing and fermentation process of grapes for winemaking [18]. Another by-product is the stalk, which is the woody part of the bunch obtained from the destemming operation [19,20,21,22,23,24,25]. Wine lees are the residue that forms on the bottom of wine containers after fermentation and during storage [20,26,27,28,29,30,31,32]. Finally, the vine shoots result from the pruning of the vine [33]. All these oenological by-products are rich in functional compounds, thus can be exploited in the food and pharmaceutical fields [34]. Table 1 summarises the functional compounds in the different oenological by-products.

Several studies have been aimed at the enhancement of oenological by-products by introducing them in the food production process, since they are rich in bioactive molecules. In particular, wine lees were added to ice cream, yogurt, cereal bars, fish burgers, bread, and fermented sausages [26,55,56,57,58,59,60], while grape marc was added to various baked goods such as muffins, breadsticks, biscuits, and bread. A further research study highlighted the addition of grape seed flour to bakery products [61]; on the contrary, the literature did not show studies about the use of stems in these products.

Therefore, the addition of oenological by-products to baked goods determines the improvement in nutritional properties, so that they can be classified as functional foods. Functional food is consumed regularly in such a way that it is defined as an integral part of a standard diet; in addition to the basic nutritional functions, it must have beneficial effects on human health by reducing the risk of chronic disease [62], and the functionality must be scientifically proven [63]. The new market requirements, influenced by the increased awareness of the close analogy between healthy eating and psycho-physical well-being, can be met by offering functional foods [64]. In addition, the increasing attention of consumers to healthy food is due to the occurrence of allergies, intolerances, as well as the potential risk of food poisoning [65,66].

### 2.2. Consumers’ Acceptance of Innovative and Functional Foods

The success of functional foods, especially if derived from by-products, depends on consumer acceptance [67]. Indeed, the need to assess consumers’ acceptance towards innovative technologies and functional foods has emerged due to the rejection of new applications [68]. In addition, agri-food industries can survive only if they respond efficiently to consumers’ demand [69].

Several factors influence consumer acceptance towards food technologies and innovations. For example, the consumer’s orientation towards natural foods identified between local and organic products is well known [70], but this trend translates into a reluctance towards innovative food technologies. In fact, the refusal even to taste unknown foods was defined as food neophobia and this affects food choices with a significant impact on diets [71]. In addition, food neophobia may be the result of social distrust [72]. Several studies observed how age can influence neophobia; in fact, it emerged that there was a higher neophobic tendency of adults compared to younger consumers [72,73,74,75]. The food acceptability of consumers towards the purchase of functional foods is also influenced by familiarity with the functional ingredient [76] added in fortified food and the greater probability that it derives from nature [77]. Taste is also a variable influencing the innovative products’ consumption [78]; in fact, many studies claimed that consumers were reluctant to purchase functional foods if their taste performance was compromised [74,79,80,81,82], although other authors highlighted a better taste acceptance of consumers when functional foods had concrete health benefits [83]. Risk perception also plays a key role in the acceptance of functional foods. In this regard, incorrect communication of actual risks would reduce the willingness to accept functional foods [84]. Temesi et al. [85] defined “Perceived Health Correspondence” as a new factor influencing the consumer’s perception of functional ingredients.

In addition, each consumer is influenced by the cultural environment in which they live, by their political, social, and ethical values, but also by institutional context [86]. For example, several studies highlighted the predisposition of many consumers to pay an increased price when the food process is environmentally friendly [87,88]. Indeed, the increase in the consumption of organic products is associated both to the perceived healthy effects of organic foods and to the lower environmental impact that the production process implies compared to conventional foods [89]. In particular, the increased propensity of women to buy organic products has emerged because they are more attentive to food safety and health issues [90,91]. Following the awareness of environmental pollution, a new consumer figure was defined: the ethical consumer. This consumer, through their purchasing choices, expresses responsibility towards society [92]. Preferences are conditioned by other individual factors such as age, gender, marital status, employment, geographical area, income bracket, and educational level [93,94]. Valid strategies to increase the demand on the market for food products enriched with food by-products include the use of clear labels [95] and the presence of ad hoc food certification [96,97,98,99].

Several studies evaluated the consumers’ acceptance of functional foods. Van Kleef et al. [100] highlighted the greater propensity of consumers to accept the enrichment of healthier and more natural foods (such as yogurt and juices) than more treated ones (e.g., chewing gum, chocolate and ice cream). Even Verbeke et al. [101] found more acceptance of cereals enriched in fibre than juices fortified with calcium, probably due to the combination juice–calcium being considered less healthy and natural compared to cereals–fibre. The results of the study by Bech-Larsen et al. [102] agreed with those by van Kleef et al. [100] and Verbeke et al. [101], as consumers preferred the enrichment of natural foods such as yogurt compared to spreadable creams. However, the literature reported few studies that were aimed at analysing the consumer’s willingness to accept foods enriched with oenological by-products. This is because the field of research is new, but it is also due to the small number of developed food products tested [103,104,105].

## 3. Materials and Methods

### 3.1. Data Collection

A questionnaire was set up to collect the variables to be analysed and was distributed through the Google Forms platform; this was a voluntary survey and respondents could leave the survey at any time. This provided a convenience sample of 250 Italian people aged from 18 to over 50 years old, who were invited to fill in the questionnaire in February 2021. We opted to use a sample of convenience consumers, since data collection is less expensive and requires less time compared to data collection performed using other sampling methods [106]. Before submitting the questionnaire to the participants, a pilot survey of 20 people was carried out to validate it. The questionnaire included a preface to inform the participants about the health and environmental benefits of the bakery products enriched with by-products from the oenological industry. The initial part aimed to investigate the knowledge of these products, the frequency of consumption of organic products, and the propensity to purchase bakery products with beneficial effects on human health and the environment. In the subsequent parts, the respondents were asked questions about their socio-demographic characteristics. Basically, the answers were provided using a 5-point Likert scale, because it enables us to obtain a precise grade of agreement or disagreement of the participants and a high level of reliability. In addition, the Likert scale is easy for participants to understand and compile the questionnaire [107]. The use of the 5-point scale is due to the higher response rate and because it appears to be less confusing, as stated by Bouranta et al. [108]. In fact, the results of the study of Preston et al. [109] stated that the scales at five, ten, and seven points collected a greater preference for ease of use. In addition, Colman et al. [110] observed a preference of participants for the odd answer categories over even categories; this is due to the possibility of being able to line them up in a neutral zone. The sample statistics and the complete set of variables are reported in Section 4.1 and Section 4.2.

### 3.2. K-Means Clustering

Cluster analysis is a multivariate statistical technique created by the biologists Sokai and Sneath in 1963 for performing biological classifications [111]. Cluster analysis refers to the application of algorithms aimed at clustering objects based on intrinsic characteristics or perceived similarity [112]. This technique does not require the use of category labels that have already been used in the past; therefore, the absence of category information makes it possible to distinguish the grouping of data from the discriminating analysis [112,113]. In this research, the K-means algorithm was applied; it partitions the data set into K-clusters with the dual objective of making each cluster as compact as possible and the K-clusters as separated as possible. K-means clustering aims to partition the data set into K-clusters (K needs to be specified by the user) in which each observation belongs to the cluster with the nearest mean [114]. According to Park and Choi [115] and Das et al. [114], the application of k-means clustering was carried out according to the following procedure: (1) select K centroids (K rows chosen at random); (2) randomly select K objects from the data set as the initial cluster centres or means; (3) assign each data point to its closest centroid, based on the Euclidean distance between the object and the centroid; (4) for each of the K clusters, update the cluster centroid by calculating the new mean values of all the data points in the cluster. The centroid of a K cluster is a vector of length p containing the means of all variables for the observations in the K cluster, while p is the number of variables; (5) iterate steps 3 and 4 until the cluster assignments stop changing or the maximum number of iterations is reached. The k-means clustering is used in this research because it enables us to identify groups of individuals who tend to be homogeneous according to the variables that can be collected using a specially prepared questionnaire [112]. Furthermore, the k-means clustering has been widely applied in several pieces of research investigating consumers’ acceptance towards different foods [116,117,118,119]. Indeed, the cluster creation is useful for identifying groups of consumers that share similar characteristics in terms of needs to be satisfied [120,121,122]. After the application of k-means clustering, the Bonferroni post hoc test was carried out to check whether the clusters differed significantly from each other with respect to the variables [123].

## 4. Results

### 4.1. Sample Statistics

The highest respondents’ quota was observed in southern Italy (71.0%), followed by shares in northern (22.0%) and central Italy (7%). Moreover, 84.5% of the respondents came from the urban centre, while the remaining sample lived in rural areas. In total, 36.8% of respondents had a family of four members, followed by three (28.5%), five (10.8%), two (17%) and one (6.9%) members. The socio-economic characteristics of the sample, which are used as variables for the k-means clustering, are reported in Table 2. Most of respondents were women (59%), while 41% were men; the predominance of the female gender is frequent in the scientific literature, due to the greater responsibility that women have in shopping than men [124,125]. Regarding the age of respondents, 60% were between 18 and 30 years old, about 29% were between 31 and 50 years old, while 11% were over 50 years old. Regarding the level of education, most of the respondents held a high school degree (54%), followed by graduates and postgraduates (43.9%) and individuals with a compulsory education level (2.1%). An over-representation of respondents with a higher level of education is in line with the study by Meyerding et al. [126], who investigated the preferences of Germans towards superfoods in bread. Finally, the majority of respondents (40.7%) have a monthly household income between 1200 and 2000 euros, while 36% have an income between 2100 and 4000 euros, 13.6% of respondents earn more than 4100 euros, and 9.7% have an income of less than 1100 euros per month. It is to be noted that the geographic and age distributions of the sample, as well as the distribution related to the socio-economic features reported in Table 2, are derived from the use of a convenience sample, and particularly from the “snowball sampling”. According to Baltar and Brunet [127], snowball sampling is a useful approach in exploratory, qualitative, and descriptive research, especially in those studies where respondents are few in number or a high degree of trust is required to initiate the contact (e.g., hard to reach/hard to involve population). When snowball sampling is carried out by means of social networks, it can be defined as “virtual snowball sampling”.

Table 3 shows the statistics about the consumers’ information and preferences used as variables. In particular, data analysis showed that 59.3% of respondents have never heard of foods enriched with wine by-products to improve nutritional characteristics and shelf-life; conversely, 28.1% of the participants have heard about these foods but never found them on the market; 6.7% have seen these products on the market; and 2.4% have found them on the market and purchased them occasionally. Finally, only 3.5% know these foods and have already appreciated them previously. As explained by Qaim et al. [128], knowledge is an enabling element of fundamental importance for healthy agri-food systems and serves to create information for improving productivity, profitability, reliability, and resilience.

Consumers were also asked to attach importance to the characteristics of bread enriched with wine by-products. Most consumers have identified naturalness as important and absolutely important (94%); this is closely associated with the high frequency of consumption of this food. Therefore, the naturalness of bread is of extreme importance for most of the respondents, while only 6% consider this aspect medium important, unimportant, and not at all important. The naturalness of the food is, together with the freshness and minimal processing, the most requested attribute for consumers [129]. In addition, the study by Coderoni et al. [99] showed a positive correlation between the level of education and the propensity towards the naturalness of food.

As for the health aspects, 22.8% of the respondents attributed fundamental importance to the characteristics of enriched bread with health benefits, while 47% and 22.1% considered this attribute to be important and of medium importance, respectively. On the contrary, there is a very low share of consumers who think that health aspects are not important. Indeed, several studies have shown that the increased propensity to consume functional foods comes from consumers concerned about their health and diet [130,131]. In addition, these results are in agreement with the research by Coderoni et al. [99], who stated that most participants were inclined to buy foods enriched with by-products with a reduced environmental impact and health benefits.

The questionnaire asked also how the possible environmental impact of enriched bread production and consumption affected consumers’ choice. In this regard, the results showed that most consumers (63%) considered this aspect important and absolutely important, 24% considered it to be of reasonable importance, while 13% attributed little or no importance to it. Therefore, most of the sample assumed a behaviour defined as “ecological”, which can be influenced by ethical aspects of the consumer or external factors, such as ecological policy constraints [132]. Several studies observed a greater propensity of young people, women, and consumers with higher levels of education to accept “environmental friendly” food [132,133,134,135,136,137].

Regarding the frequency of consumption of organic foods and products, 9.8% of the respondents buy organic products regularly such as detergents, cosmetics, and clothing, while 15.10% consume organic foods regularly. About one third of respondents (33.3%) consume only specific categories of organic food and 35.1% occasionally consume organic products, while 6.7% do not consume any type of organic food and products. The propensity to purchase organic foods and products may be associated with environmental concerns [138].

Technophilia, defined as the willingness of consumers to buy foods obtained from innovative technologies, was also analysed [139]. The survey showed that only a small proportion of consumers (11%) were not prepared to accept new technologies under any circumstance; on the contrary, a high quota of consumers (30.5%) expressed a willingness to accept innovative technologies only if the safety features of the product were guaranteed by a certification; overall, 21.6% of participants would be willing to accept these technologies only with guarantees from the manufacturer, 25.9% only after a personal retrieval of information, while 11% would accept them in any case. Therefore, the sample is characterised by distrust towards food technologies, unless there are guarantees provided by certification boards and manufacturers.

In addition, the respondents were asked about their willingness to pay (WTP) for enriched bread with wine by-products. This showed that the most significant share of consumers (45.6%) would be willing to pay 10% more for this product, while 22.8% would pay for enriched bread only if it was sold at the price of conventional bread; another 22.8% would instead be willing to pay 20% more, and only 2.8% would pay 30% more. Only 6% would not pay for enriched bread in any case. Consequently, more than 70% of respondents would be willing to pay a premium price for this enriched bread.

An investigation of the willingness to accept modifications of traditional foods to improve their nutritional characteristics and increase their shelf-life showed that 33.7% of consumers are willing to accept these interventions, while 19.3% declared acceptance only to improve nutritional characteristics, and 6.7% only to prolong the shelf-life. Additionally, 19.6% of consumers stated that traditional foods must not be modified, and 20.7% would accept changes provided that the tradition remains unchanged.

Regarding the willingness to buy enriched food based on the nutritional declarations on the label, 34% of participants stated a propensity to purchase an enriched food if it was accompanied by a certification and an explanatory label of the benefits, 28.1% only with a comprehensive explanation of the benefits on the label, 22.8% after personally investigating the benefits, and 6.3% even without explicit statements on the label. Only 8.8% of respondents were not influenced by the label.

Consumers’ food choices are heavily influenced by taste [78]. In fact, 10.2% of respondents said that they would never accept a bread that differed in terms of taste from conventional bread, even if it was beneficial for health; 22.8% would only accept it if it had the same taste as the conventional bread; and the most significant percentage of consumers would be willing to accept a slightly different taste provided that the bread is actually beneficial for health (55.4%). Consumers willing to buy enriched bread even if it has a taste completely different from the traditional one are equal to 5.6% of those surveyed, while 6% would buy it regardless of the taste.

The willingness to accept enriched bread was also investigated. In total, 33.0% of survey participants were willing to buy enriched bread only if it retained the same organoleptic characteristics as conventional bread, while 30.0% would be willing to accept the organoleptic features of enriched bread, provided that they are clearly declared on the label. Overall, 17.0% of participants were inclined to buy functional bread only if these characteristics differed slightly from the not-enriched product, while 12.0% would not buy it anyway. Only 8.0% would always accept enriched bread.

### 4.2. Cluster Analysis

**Figure 1:**
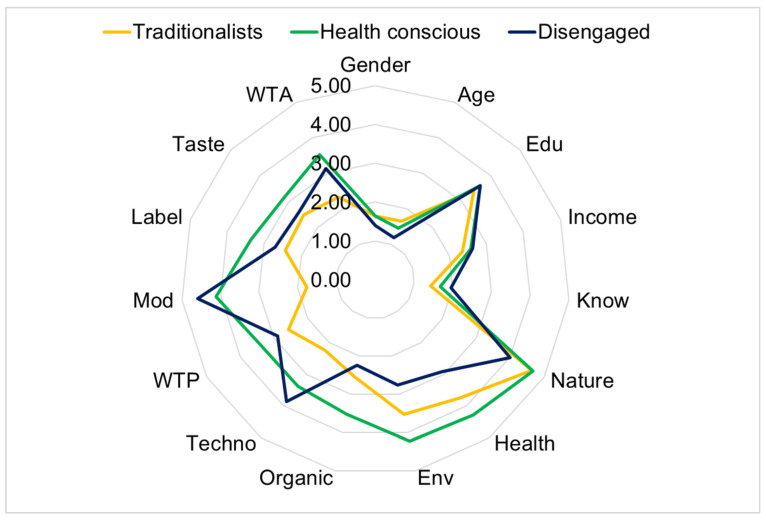
The radar chart showing the three clusters of consumers.

The final centroid values enabled us to determine the “labelling” of the clusters, and thus the assignment of the consumers to a specific cluster. The following three clusters of consumers were identified: (1) the “traditionalists”; (2) the “health-conscious”; (3) the “disengaged”. The features of these clusters are shown in Figure 1 and Table 4, and are described hereafter.

The first cluster is called “traditionalists” and includes 107 consumers, corresponding to 42.8% of the sample. These consumers are mainly women, with the lowest income and age among the three clusters, and a low average level of education. The definition “traditionalists” is mainly due to the reluctance towards innovations in food production processes and the modification of traditional products. These consumers are also reluctant to accept unconventional tastes, and show an average interest in the aspects of naturalness and health beneficial effects associated with enriched bread consumption. However, the cluster of traditionalists does not know about bread enriched with wine by-products; thus, they are the most reluctant consumers. In addition, they show a lower WTA and WTP than the other two clusters, which may be due to their moderate focus on health, the environment, and reluctance to accept this food even though the label fully explains its nutritional benefits. These consumers have a low awareness of environmental sustainability and do not consume organic products regularly. The traditionalists, therefore, are consumers with a conservative behaviour, in such a way that the variation in the characteristics of conventional foods prevents their acceptance of food innovation [140].

The second cluster is made up of 95 “health conscious” consumers, who correspond to 38% of the sample. They mainly consist of women, and are mostly young and with a high level of education and income. The opposite results were observed by Annunziata and Vecchio [93], who identified older people as responsible consumers. In this second cluster, the consumers are more attentive to the naturalness of bread, with a frequent consumption of organic products, and are very attentive to health and environmental aspects. These findings agree with those of Niva et al. [141], who observed the correlation between sustainable food consumption and healthy eating, since the interest in environmental sustainability influences the eating behaviour. In addition, in some cases, there may be a link between attention to health and environmental concerns [142]. Moreover, the health conscious are also quite inclined to accept food obtained with innovative technologies, as well as modifications of traditional foods to improve their nutritional characteristics and increase shelf-life. The antithesis existing between tradition and innovation is known; however, the claim of the health conscious was favourable in both aspects [143]. In fact, as explained by Cavaliere et al. [144] in their research about the mismatch between food sustainability and the acceptance of innovation technologies among millennial students, the consumer’s focus on sustainable aspects could encourage the acceptance of food technologies. In this second cluster, the consumers are the readiest to accept a different taste of bread if this provides health benefits. They know about the enriched bread; however, they are inclined to accept this food if the label explains the nutritional benefits. In fact, as explained in the study by Annunziata and Vecchio [145] on the consumers’ perspective on functional foods, consumers are willing to purchase enriched foods if they can associate them with health properties that are absent in conventional foods, and thus they are willing also to sacrifice the pleasantness of taste and pay a higher price. Indeed, consumers choose more consciously with sufficient knowledge, showing a higher level of acceptance towards functional foods [142]. Finally, the consumers in this cluster declared the highest WTA and WTP.

Forty-eight consumers belong to the “disengaged” cluster (equal to 19.2% of the sample), half men and half women, mostly young and with the highest level of education and income. They do not pay a lot of attention to the naturalness of bread nor to its environmental sustainability and health benefits, and they do not consume organic foods even though they know about them. Moreover, these consumers are more likely to accept innovative technologies, and attach fundamental importance to the traditional nature of food, but are quite inclined to accept changes in taste if nutritional aspects and shelf-life are improved, especially if food characteristics are well explained on the label. They declared an average WTA and WTP.

The results of the Bonferroni post hoc test are shown in Table 4, and highlighted significant differences among the variables within the clusters, as indicated by different letters for each variable. In particular, the highest difference within the consumers’ clusters is determined by the importance of the beneficial effects of bread consumption (Health), by the importance associated with product characteristics having an impact on the environment (Env), by the willingness to buy food obtained with innovative technologies (Techno), and by the willingness to accept the modifications of traditional foods to improve their nutritional characteristics and increase shelf-life (Mod). On the contrary, there is only one variable showing no significant difference, namely the monthly household income (Income). This result pointed out that the acceptance of this enriched bread is not influenced by the consumers’ socio-economic features, but it is related to consumers‘ sensitivity.

## 5. Discussion

The results of this study highlight the lack of knowledge about the enriched bread by consumers (in particular the cluster of traditionalists). In addition, consumers (especially those belonging to the health conscious group) attach great importance to the naturalness of bread because it represents a significant part of the expense for purchasing [146] and determines a careful attitude by the consumer in relation to the possible health beneficial effects. Although the study proposes an innovative food, which should possess greater appreciation and a higher price, it is at the same time a common food consumed daily. Therefore, most consumers are not willing to pay a price 10% higher than the price of conventional bread, regardless the monthly household income and the cluster of consumers. A higher WTP for functional foods was found by Mirosa et al. [147] in the Chinese population (40%) and by Menrad et al. [148] in the European consumers (between 30 and 50%) for functional foods. However, the WTP could increase for consumers who are suffering from a disease [149]. On the contrary, the research by Nazzaro et al. [140] pointed out the willingness of more than half of the consumers to pay higher prices for the purchase of innovative ‘panettone’ compared to the traditional one. The health conscious consumers are willing to accept a different taste in favour of the health benefits, and show higher WTA and WTP than the consumers belonging to the other clusters. The disengaged consumers do not pay attention to health and the environment; however, they show an intermediate WTA and WTP. This reflects a market situation in which the preferences are not homogeneous, and the socio-economic variables are not significant in the definition of the WTA/WTP. Contrary to health conscious consumers, there are traditionalist consumers who do not know this type of bread; thus, they are also the most reluctant in terms of acceptance. The lower WTA can also be traced back to their moderate attention to both health and the environment, but also to their unwillingness to accept this food even if the label fully explains the benefits. Therefore, encouraging this cluster of consumers to accept new technologies and innovative products is necessary in order to study the factors that predispose them to acceptance or scepticism towards food innovation [150,151,152,153]. In addition, an understanding of the benefits associated with the consumption of this food should be facilitated.

The study also highlights how some attributes were recognized as more important than others for the consumers’ characterisation. In particular, taste is one of the attributes considered most important for the consumers, since there is a positive correlation between an optimal taste and the acceptance of the enriched bread. Several studies confirmed the importance of taste to the consumer [154,155]; in fact, a greater influence of taste than of the health benefits was seen [81,85,156]. For this reason, it is necessary to pay great attention to taste during the formulation of an innovative product in order for it to be acceptable [80].

In addition, technophilia influences consumers’ choices. Almost all the respondents have declared that they are willing to accept these food technologies, provided they are guaranteed by the producer or by a certification (seeking for reassurance), as the introduction of these may lead to difficulties in assessing the risks and benefits associated with them [157]. The study by Cavaliere et al. [142] highlighted that consumers who were very attentive to sustainability could not accept the contributions of science and technology to achieve a more sustainable society; this concept contrasts with the results of this study in which there was a positive correlation between sustainability and trust in technologies. Moreover, the clusters of disengaged and health conscious consumers are willing to accept food modifications if they do not alter the traditionality of the enriched bread. In fact, an optimal innovation process is one that follows tradition and improves it with the modern technologies available [158]. Only traditionalists are reluctant to accept changes in conventional foods, and these are mainly women with a lower-than-average income, living in rural areas, and with a low level of education, in line with the study by Vanhonacker et al. [159].

The last variable considered crucial is the consumption of organic foods and products. In this regard, the consumers of these foods and products (particularly the health conscious) are more reluctant to accept the addition of substances to the food itself, and for them the food naturalness is of fundamental importance. Indeed, the consumption of organic foods is associated with environmental and health benefits, and with a better taste than conventional foods [138]. Therefore, the bread enriched with wine by-products could be accepted by the health conscious consumers because they are very attentive to environmental sustainability, achievable through the transformation of food waste into functional substances to be added to bread. These consumers are also prone to accepting changes aimed at improving food nutritional characteristics and shelf-life.

## 6. Conclusive Remarks

This research explored the attitude of consumers towards innovative functional foods enriched with oenological by-products. The cluster analysis showed the characterisation of consumers into three clusters with different features: The traditionalists are the consumers reluctant to accept innovative technologies and show little interest in the health aspects and environmental sustainability. The health conscious cluster of consumers is the readiest to accept enriched bread because these consumers are inclined to accept healthy products with less environmental impacts, and would accept the modification of traditional products for the improvement in nutritional characteristics and shelf-life. Finally, disengaged consumers show no interest in environmental and health issues, but are inclined to accept changes to improve nutritional and shelf-life aspects.

Future studies will focus on the study of the variables considered, prompting the most reluctant consumers to accept enriched bread. Therefore, winning marketing strategies should be studied to encourage ‘traditional’ consumers to appreciate the naturalness and beneficial effects that bread enriched with by-products can have on human health and the environment. This goal can be achieved with an exhaustive explanation about the health benefits of this innovative product, highlighting also that the tradition of the production process has not been altered. On the other hand, it would be necessary to raise the awareness of disengaged consumers towards the environmental and health issues, which can be tackled by the reuse of by-products in the production cycle of bakery goods. Finally, health conscious people could be considered as “exemplary consumers” because they are sensitive to health and environmental issues in such a way that they can trigger a circular economy model.

## Figures and Tables

**Table 1:** The functional compounds in the oenological by-products.

Functional Compounds											
By-Products	TDF (g/100 g)	Pr. (g/100 g)	Tan. (g/100 g)	Hem. (g/100 g)	Cel. (g/100 g)	Lig. (g/100 g)	Pec. (g/100 g)	TPC (mg/g)	TAC (mg Cyn 3 glu/g)	Toc. (mg/kg)	Refs.
Wine lees	29.4–82.3	7–20.3	n.a.	28.8	30.0	44.4	12.4–12.8	16.4–254.0	383.1	n.a.	[20,27,28,29,30,31,32,34]
Grape stems	71.4	6.1–8.0	6.4–16.0	14–35.3	14.6–30.0	17.3–47.3	31.1	128.0–215.0	n.a.	n.a.	[19,20,21,22,23,24,25]
Grape pomace	17.3–58.0	5.4–15.5	2.2	22.6	7.2	19.4–47.3	3.4–31.4	11–90.2	7.4–88.4	n.a.	[20,24,25,35,36,37,38,39,40,41,42]
Grape seeds	58.8	6.3–11.0	6.4	n.a.	n.a.	n.a.	n.a.	0.1–0.2	n.a.	67.7–290.5	[38,39,42,43,44,45,46]
Vine shoots	n.a.	4.0–5.3	n.a.	n.a.	n.a.	49.0	n.a.	5–225.0	n.a.	n.a.	[47,48,49,50,51,52,53,54]

Notes: n.a. = data not available; TDF = total dietary fibre; Pr. = protein; Tan. = tannins; Hem. = hemicellulose; Cel. = cellulose; Lig. = lignin; Pec. = pectin; TPC = total phenol content; TAC = total anthocyanin content; Toc. = tocopherols. Refs. = references.

**Table 2:** Socio-economic variables of the sample. The codes are in square brackets.

Gender [Gender]	% of Respondents
Female	59.0
Male	41.0
**Age [Age]**	
From 18 to 30 years old	60.0
From 31 to 50 years old	29.0
Over 50 years old	11.0
**Education level [Edu]**	
Compulsory school	2.1
High school	43.9
University degree or postgraduate	54.0
**Monthly household income [Income]**	
Less than 1100 euros per month	9.7
From 1200 to 2000 euros per month	40.7
From 2100 to 4000 euros per month	36.0
Over 4100 euros per month	13.6

**Table 3:** Variables on consumers’ information and preferences. The codes are in square brackets.

Knowledge of Consumers on Foods Enriched with Wine By-Products to Improve Organoleptic, Nutritional, and Sustainability Characteristics [Know]	% of Respondents
No knowledge	59.3
Vaguely known but not found on the market	28.1
Found on the market	6.7
Found occasionally on the market and purchased	2.4
Known and appreciated before	3.5
**Emphasis on the naturalness of bread enriched with wine by-products [Nature]**	
Not at all important	0.7
Unimportant	0.4
Medium important	4.9
Important	31.0
Absolutely important	63.0
**Importance of the beneficial effects of bread consumption [Health]**	
Not at all important	1.4
Unimportant	6.7
Medium important	22.1
Important	47.0
Absolutely important	22.8
**Importance associated with product characteristics having an impact on the** **environment [Env]**	
Not at all important	3.0
Unimportant	10.0
Medium important	24.0
Important	44.0
Absolutely important	19.0
**Frequency consumption of organic foods and products [Organic]**	
Never	6.7
Occasionally	35.1
Only certain types of organic foods and products	33.3
Regular consumption of organic foods	15.1
Regular consumption of organic products (cosmetics, detergents, clothing)	9.8
**Willingness to buy food obtained with innovative technologies [Techno]**	
No willingness to buy	11.0
Only with a certification	30.5
Only with manufacturer’s guarantees	21.6
After personal retrieval of information	25.9
Willingness to buy in any case	11.0
**Willingness to pay enriched bread with wine by-products [WTP]**	
No willingness to pay	6.0
At the same price as conventional bread	22.8
10% more than conventional bread	45.6
20% more than conventional bread	22.8
30% more than conventional bread	2.8
**Willingness to accept modifications of traditional foods to improve their nutritional characteristics and increase shelf-life [Mod]**	
Traditional foods must never be modified	19.6
They can be modified without altering them traditionality	20.7
Only to improve nutritional characteristics	19.3
Only to prolong the shelf-life	6.7
To improve nutritional characteristics and shelf-life	33.7
**Willingness to buy enriched food if it shows nutritional declarations on the label [Label]**	
Would not affect the choice	8.8
Yes, only if guaranteed by a certification	34.0
Yes, only with a comprehensive explanation of the benefits on the label	28.1
Yes, after having personally researched the nutritional benefits	22.8
Yes, even without the statements of nutritional benefits on the label	6.3
**Propensity to prefer enriched bread with a positive effect on health even if it is less tasty than conventional bread [Taste]**	
No, in any case	10.2
Yes, only with the same taste as the conventional bread	22.8
Yes, even with a slightly different taste is beneficial for health	55.4
Yes, even with a different taste	5.6
Yes, absolutely	6.0
**Willingness to accept enriched bread with wine by-products [WTA]**	
No willingness to accept	12.0
Yes, only if the organoleptic features are quite similar to the conventional bread	33.0
Yes, even with a slightly different organoleptic features	17.0
Yes, with different organoleptic features if they are clearly declared	30.0
Yes, in any case	8.0

**Table 4:** The final centroid values of the variables according to each cluster and the results of the Bonferroni post hoc test.

Variables	Clusters		
	Traditionalists	Health Conscious	Disengaged
Gender	1.64 a	1.63 a	1.40 b
Age	1.64 a	1.45 a,b	1.19 b
Edu	3.42 a	3.62 b,c	3.63 a,c
Income	2.36 a	2.58 a	2.63 a
Know	1.44 a	1.68 a,c	1.96 b,c
Nature	4.64 a	4.67 a	4.00 b
Health	3.74 a	4.29 b	2.92 c
Env	3.53 a	4.23 b	2.77 c
Organic	2.55 a,c	3.53 b	2.25 c
Techno	2.22 a	3.38 b	3.88 c
WTP	2.57 a	3.39 b	2.90 a
Mod	1.78 a	4.12 b	4.58 c
Label	2.44 a	3.36 b	2.71 a
Taste	2.49 a	3.16 b	2.63 a
WTA	2.33 a	3.53 b,c	3.13 c

Note: according to the Bonferroni post-hoc test, significant differences among the variables within the clusters are highlighted by different letters.

## Data Availability

The data presented in this study are available on request from the corresponding author.
